# Deciphering the role of extrachromosomal circular DNA in adipose stem cells from old and young donors

**DOI:** 10.1186/s13287-023-03575-2

**Published:** 2023-11-28

**Authors:** Sen Ren, Du Wu, Xiaoyong Shen, Qian Wu, Chengcheng Li, Hewei Xiong, Zhongwei Xiong, Rui Gong, Zheng Liu, Wei Wang, Jincao Chen

**Affiliations:** 1 Department of Neurosurgery, Zhongnan Hospital of Wuhan University, Wuhan, 430071 China; 2 Hospital of Stomatology Wuhan University, Wuhan, 430079 China; 3 Department of Hand Surgery, Union Hospital, Tongji Medical College, Huazhong University of Science and Technology, Wuhan, 430022 China; 4 Department of Emergency Surgery, Union Hospital, Tongji Medical College, Huazhong University of Science and Technology, Wuhan, 430022 China

**Keywords:** Adipose stem cell, Aging, Extrachromosomal circular DNA

## Abstract

**Background:**

The functional impairment of adipose stem cells (ASCs) during aging limits their clinical transformation. Studies have shown that extrachromosomal circular DNAs (eccDNAs) are associated with tumor progression and cell aging, but the roles of eccDNAs in ASCs remain unknown.

**Method:**

We conducted Circle sequencing (Circle-seq) to identify eccDNAs in ASCs isolated from young and old donors. The differentially expressed eccDNAs were calculated, annotated and validated via polymerase chain reaction.

**Results:**

Thousands of eccDNAs were identified and comprehensively characterized. Most of them were GC-rich, < 1000 base pairs in size, and were enriched on chromosome 19 and 17 with a high density of Alu elements and genes, 2 kb upstream/downstream of genes and satellites. In total, 3025 eccDNAs were differentially expressed among the two ASC groups. Conjoint analysis of the Circle-seq results and previous RNA-seq results revealed that 73 eccDNAs and 55 genes exhibited the same differential expression between the two groups. KEGG and GO analyses revealed that genes encoding differentially expressed eccDNAs were enriched for cell adhesion, cellular senescence and TGF-β receptor signaling pathway. We also found that aged ASCs exhibited loss of eccDNAs, including CAMK2G ^(chr10: 75577899-75578176)^, TRABD2B ^(chr1: 48305638-48307008)^ and TRABD2B ^(chr1: 48305425^^-48307091)^.

**Conclusion:**

In this study, we elucidated the first eccDNA profile relating to ASCs and demonstrated that three eccDNAs are lost in aged ASCs, which may be potential biomarkers of stem cell aging and valuable targets for stem cell rejuvenation.

**Supplementary Information:**

The online version contains supplementary material available at 10.1186/s13287-023-03575-2.

## Background

Mesenchymal stem cells (MSCs) are powerful tools for tissue repair and can be isolated in almost all organs, especially adipose tissues [1]. Adipose stem cells (ASCs) are harvested in a less invasion manner accompanied with an esthetic benefit; these cells proliferate rapidly and have immunoregulatory properties, making them useful for various processes including wound healing, nerve injury, and cardiac repair [2–4]. Increasing evidence suggests that the function of MSCs declines with aging, which limits their clinical transformation [5]. Previous studies showed that ASCs from elderly donors are associated with a high rate of senescence, exhibit a senescence-associated secretory phenotype (SASP), and have limited capacity for use in the regeneration of other tissues and organs [6, 7]. There are two strategies to overcome aging-related impairment of MSCs, either the identification of functional stem cell subpopulations, or rejuvenation and energization of aged stem cells. However, there is a lack of appropriate markers to enrich for beneficial stem cells and feasible approaches to restore aged stem cell function.

Extrachromosomal circular DNAs (eccDNAs) are novel circular DNAs that exist outside chromosomes in almost all eukaryotic cell nuclei [8].The mechanisms for generating eccDNAs remain largely unclear, but evidence suggests associations with DNA damage and repair, chromothripsis, and apoptosis [9, 10]. EccDNAs have been demonstrated to play a vital role in tumor pathogenesis, heterogeneity and therapeutic resistance, partially through the amplification of oncogenes including EGFR, MYC, and MYCN [11–13]. Moreover, eccDNAs are also recognized as valuable biomarkers for the noninvasive diagnosis and surveillance of cancer and urogenital disorders [14–16]. Interestingly, eccDNA accumulation has long been identified in aged yeast and mammalian cells [17, 18]. A recent study revealed that eccDNAs formation in aging budding yeast can be triggered by the transcription of genes that are sensitive to environmental stress [19]. Another study showed high variability in plasma eccDNAs among adult healthy mice; however, age, sex and body weight have no impact on eccDNAs [20]. Little is known about the origin, generation and function of eccDNAs either in cancer progression or age-associated diseases. Furthermore, there is a lack of research on the basic characteristics and functions of eccDNAs in stem cells, especially during aging.

In the present study, we hypothesized that the expression pattern of eccDNAs in ASCs changes during aging, which might play a role in stem cell dysfunction. To our knowledge, ours is the first study to characterize eccDNAs in ASCs isolated from three old and three young donors via Circle sequencing (Circle-seq). To test our hypothesis, the basic characteristics (number, size, GC contents, chromosomal distribution, genomic annotation and junction motif) and expression profiles of eccDNAs in two groups were calculated and compared. Surprisingly, three eccDNAs generated from two protein-coding genes were found to be depleted in aged ASCs, highlighting potential biomarkers for stem cell aging and valuable targets for stem cell rejuvenation.

## Methods

### Cell isolation, culture and identification

The collection of human subcutaneous adipose tissues was approved by the Ethics Committee at the Tongji Medical College of Huazhong University of Science and Technology. Adipose stem cells were then isolated and cultured in a medium containing Dulbecco’s modified Eagle’s medium (Cyagen Biosciences, China) supplemented with 10% fetal bovine serum (Cyagen Biosciences, China) and 1% penicillin/streptomycin. The flow cytometric analysis and multi-lineage differentiation method were used to identify adipose stem cells under passage three, following the protocols outlined in previous publications [2, 21]. The adipose stem cells were divided into two groups: young group (Y-ASC) from donors under 12 years old and old group (O-ASC) from donors over 50 years old. The basic characteristics of these two groups are described in Additional file 1: Table S1.

### Construction of eccDNA library

In this study, ASCs at passage three were utilized. The extraction, purification and library construction of eccDNAs were performed according to a previous protocol [22]. Initially, the entire genome of ASCs was extracted following the manufacturer’s instructions (Tiangen, DP304-03). The linear DNA was then eliminated by exonuclease (Lucigen, E3110K). The enzymatic activity of the exonuclease was deactivated at 70 °C heat for 30 min. Polymerase chain reaction (PCR) was then conducted to confirm the successful removal of linear DNA. The purified eccDNAs were subsequently utilized as templates for phi29 polymerase amplification (REPLI-g Midi Kit, QIAGEN, Germany). The phi29-amplified DNA was fragmented through sonication (Bioruptor), and the resulting purified fragmented DNA was employed for library construction using the NEBNext® Ultra II DNA Library Prep Kit for Illumina. The library was subsequently purified using beads, and the size distribution of the fragments was analyzed to evaluate their quality.

### Circle-seq analysis

The eccDNA library was sent for the Circle-seq data analysis (DIATRE Biotechnology, Shanghai, China). The linear eccDNA products were sequenced using NovaSeq 6000 platforms. The quality of the initial data was assessed and trimmed using Trim Galore software (https://www.bioinformatics.babraham.ac.uk/projects/trim_galore/). Then, the clean reads were aligned to the reference genome (hg19) using BWA software [23]. The identification of eccDNAs in each sample was performed using Circle-MAP software [24], and a BED file containing the genomic information of each eccDNA was generated. Finally, the eccDNAs were annotated using Bedtools software [25].

### Analysis of differentially expressed eccDNAs

The raw counts of circular DNAs were calculated using Bedtools software [25]. Then, the differential expression of eccDNAs was determined by DEGseq [26]. The significantly dysregulated eccDNAs must meet the following criteria: (1) fold changes ≥ 2 or ≤ − 2, and (2) *q* value < 0.001.

### Bioinformatics analysis

The RNA-seq data utilized in this investigation were obtained from our previously published article [27] and were deposited in the Gene Expression Omnibus (http://www.ncbi.nlm.nih.gov/geo; GSE174502). DEGseq [26] was employed to conduct the differential expression analysis of mRNAs, with fold changes ≥ 2 or ≤ − 2 and *q* value < 0.001 as the criteria. The biological function of genes was determined by GO database (http://geneontology.org) and KEGG database (http://www.genome.jp/kegg).

### Genomic annotation of eccDNAs

The genomic annotation of eccDNAs was performed following the procedures outlined in previous studies [15, 28]. Briefly, the overall populations of eccDNAs were located in the human genome (hg19), and the number of these molecules’ start position mapped to the genome elements (exon, intron, Gene2KbD, Gene2KbU, 3′UTR, 5′UTR, CpG island, Alu) was calculated. The “normalized genomic coverage” of eccDNAs in each genomic element is the percentage of molecules falling in that class of genomic elements divided by the percentage of the genome covered by that class of elements. Next, the amount of DNA reads mapped to each repetitive sequence was quantified using the BedTools software [25]. Then, the percent of reads falling in the specific repetitive element was calculated.

### Junction motif of eccDNAs

The ten bp up- and downstream sequences on the junction site of each eccDNAs were extracted using BedTools software [25]. The trinucleotide motifs of eccDNAs from two groups were analyzed using a previous method [28]. The expected frequency of each motif was calculated using BedTools [25] and displayed in WebLogo [29]. The trinucleotide motif sequences on the junction sites were then inferred from the reference genome.

### PCR and Sanger’s sequencing

The eccDNAs generated from calcium/calmodulin-dependent protein kinase II gamma (CAM2KG) and TraB domain containing 2B (TRABD2B) were visualized using IGV software [30]. The total eccDNAs in each sample were isolated and purified using the above method. Rolling circle amplification was then conducted to increase the eccDNA yield. PCR was then carried out using the PrimeSTAR® Max DNA Polymerase kit (Takara, R045A), and the primers are described in Additional file 1: Table S2. Next, the PCR products were loaded onto 1.5% agarose gels and observed using an ultraviolet luminescent image analyzer. Sanger’s sequencing was used to determine the specific sequences of each PCR product.

### Statistical analysis

All data were statistically analyzed using the Graph Pad prism v 7.0 software. The Wilcoxon test was used to compare the difference of two groups. The correlation analysis was assessed by Pearson correlation coefficients. Statistical significance was set at *p* < 0.05.

## Results

### Basic features of eccDNAs in ASCs detected by Circle-seq

**Fig. 1:**
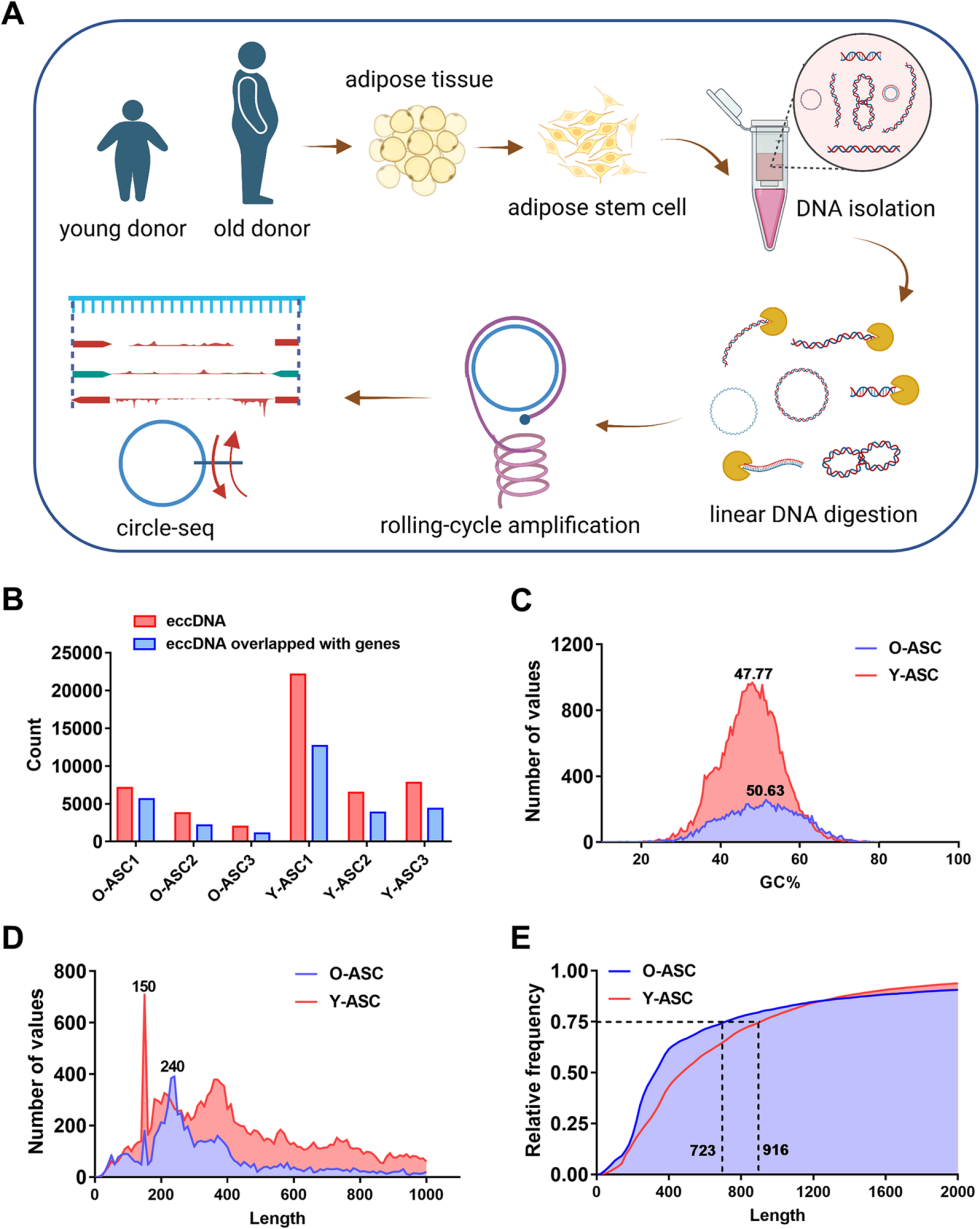
Basic features of eccDNAs. **A** The flow diagram of this study. **B** The quantification of eccDNAs in individual samples. **C** The analysis of the GC content of eccDNAs in ASCs obtained from both elderly and young donors. **D** The examination of the size distribution of eccDNAs in ASCs derived from elderly and young donors. **E** Cumulative frequency plots of eccDNAs in ASCs from elderly and young donors

**Fig. 2:**
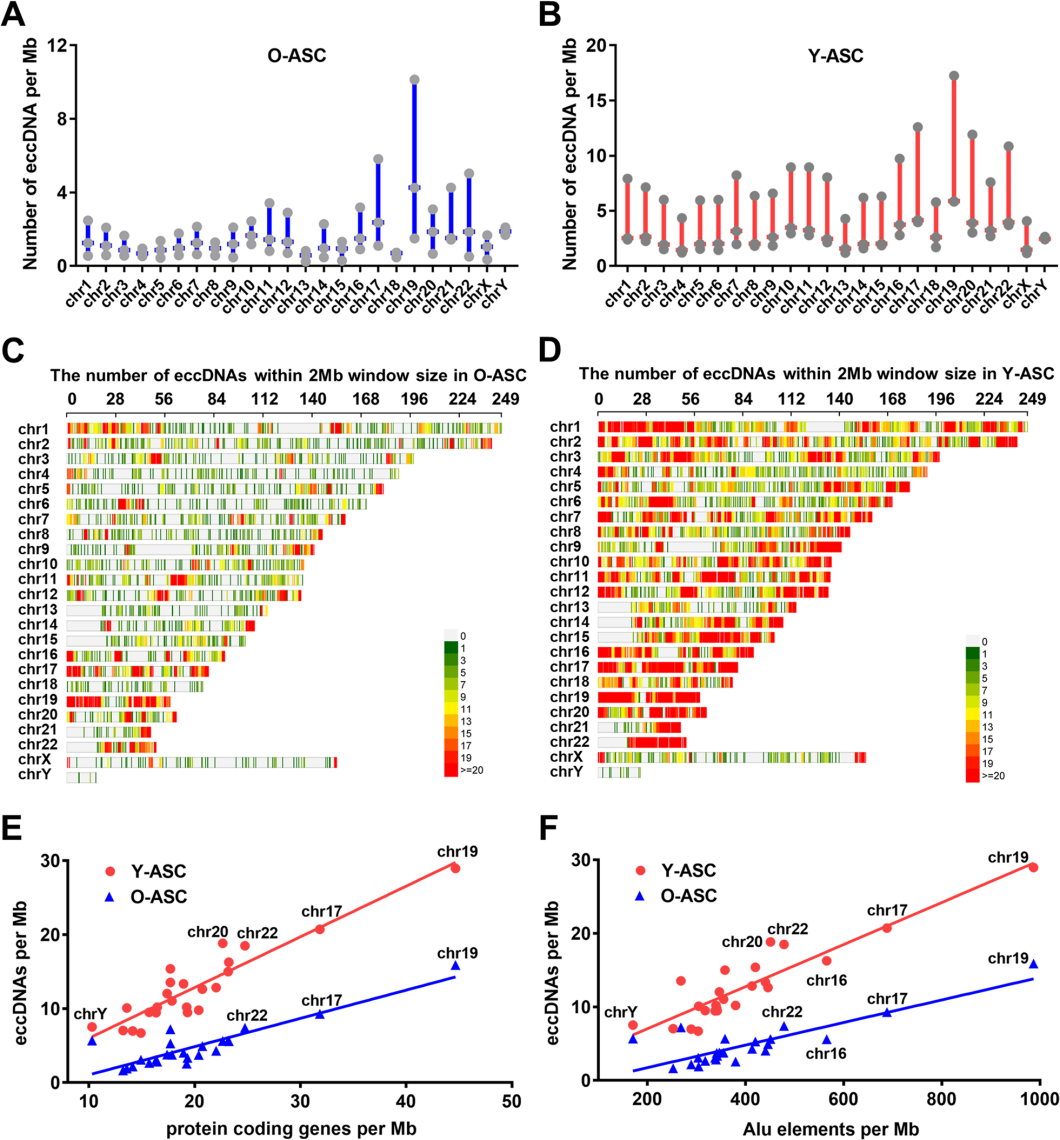
Chromosomal distribution of eccDNAs. **A** and **B** The distribution of eccDNA in both aged and young ASCs was analyzed, with a focus on the number of eccDNA per megabase (Mb) in various chromosomes. **C** and **D** Karyotype plots exhibiting chromosomal distribution of eccDNA in aged and young ASCs. **E** and **F** The positive correlation between the number of eccDNA per Mb with the number of protein coding genes per Mb, as well as the number of Alu elements per Mb

We conducted Circle-seq analysis to capture eccDNAs in ASCs isolated from young and old donors. The experimental procedure is illustrated in Fig. 1A. A total of 46,432 eccDNAs were identified in six samples by Circle-Map software. Young ASCs exhibited more eccDNAs than old ASCs. We identified 22,110, 6497, and 7803 eccDNAs, respectively, in each of the three young ASCs (Additional file 2: Data S1, Additional file 3: Data S2 and Additional file 4: S3), and 7103, 3756, and 1982 eccDNAs, respectively, in each of the three old ASCs (Additional file 5: Data S4, Additional file 6: S5 and Additional file 7: S6); most of these eccDNAs mapped with coding DNA location (Fig. 2B). Next, we found that eccDNAs from young patients had higher abundance of GC contents vs eccDNAs from old patients [mean 47.77% vs. 50.63%, respectively (Fig. 2)]. We also found that around 80% of eccDNAs were < 1000 bp in size. Strikingly, the size distribution of these eccDNAs was aggregated in four specific peaks, with the top peak positioned at 150 bp in eccDNAs from young ASCs and at 240 bp in eccDNAs from old ASCs (Fig. 2D). Furthermore, the median length of eccDNAs in young ASCs was 481 bp compared with 334 bp in eccDNAs from old ASCs. The 75% percentile for eccDNA size was 916 bp and 723 bp in ACSs from young and old donors, respectively.

### Chromosomal distribution of eccDNAs in ASCs

We next investigated the chromosomal distribution of eccDNAs from young and old ASCs by aligning them to the human reference genome (hg19). We found that eccDNAs were distributed across all chromosomes and that the largest number of eccDNAs were localized to chromosome 1 (3199 in Y-ASC and 1068 in O-ASC); chromosome Y was associated with the fewest eccDNAs (Fig. 2A, B). The enrichment of eccDNAs from each chromosome was calculated by normalizing the number of eccDNAs to the size of the respective chromosome. Interestingly, chromosome 19 was associated with the most eccDNAs per Mb, followed by chromosome 17, and chromosome X and chromosome 13 were associated with the least eccDNAs per Mb in Y-ASC and O-ASC, respectively (Fig. 2C, D). Since chromosome 19 and 17 were enriched with protein-coding genes, we analyzed the relationship between eccDNAs and protein-coding genes. The results demonstrated a positive correlation between the number of eccDNAs per Mb and protein-coding genes per Mb both in Y-ASC (*p* value < 1.00E−4, Pearson’s *R* = 0.848) and O-ASC (*p* value < 1.00E−4, Pearson’s *R* = 0.774) (Fig. 2E). This phenomenon suggests that eccDNA generation may associate with the openness of chromatin. Furthermore, chromosome 19 and 17 were enriched with Alu elements, and we also observed a positive correlation between the number of eccDNAs per Mb and Alu elements per Mb both in Y-ASC (p value < 1.00E-4, Pearson’s R = 0.822) and O-ASC (*p* value < 1.00E−4, Pearson’s *R* = 0.695) (Fig. 2F).

### Genomic annotation of eccDNAs in ASCs

**Fig. 3:**
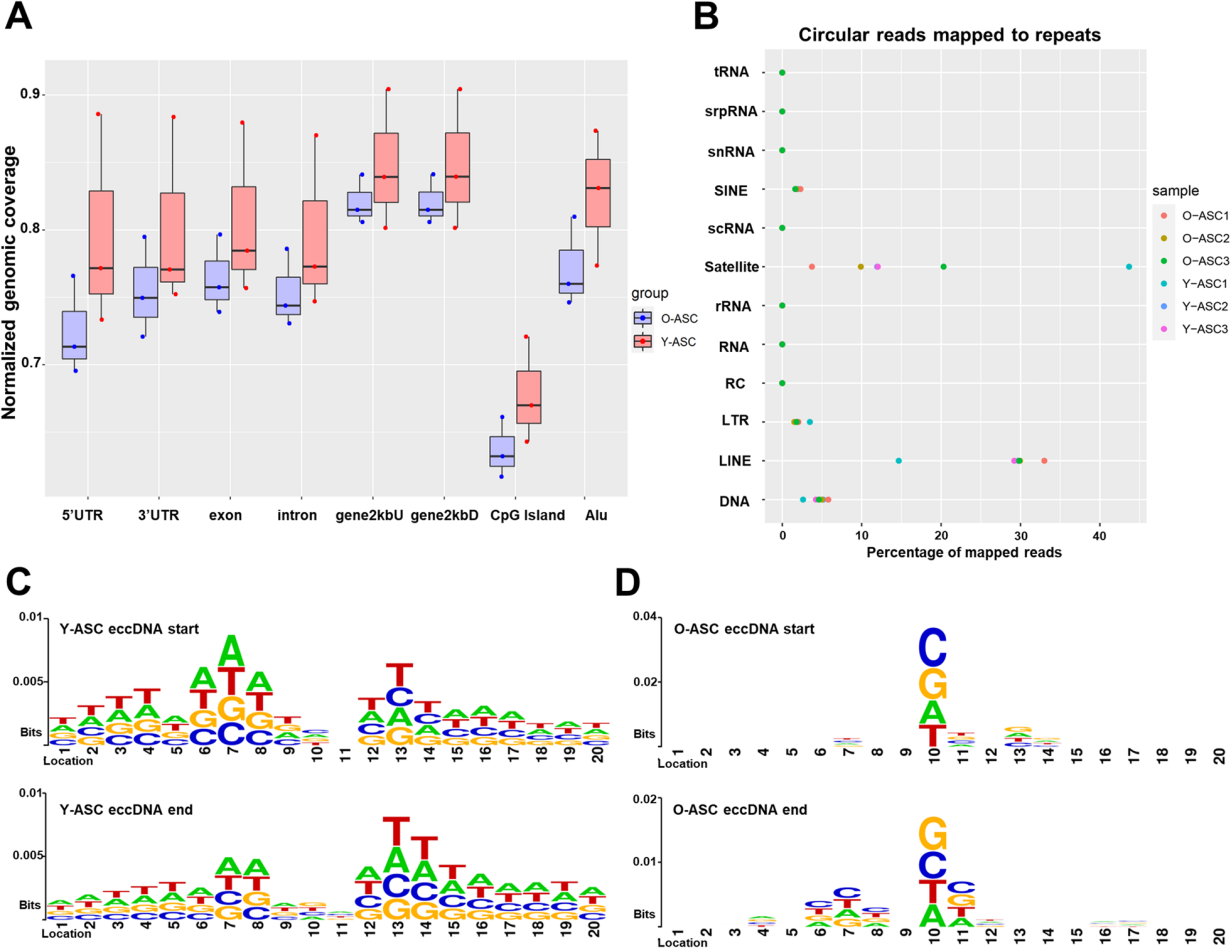
Genomic distribution of eccDNAs. **A** Distribution of eccDNAs in each genomic element; the “normalized genomic coverage” is the percentage of molecules falling in that class of genomic elements divided by the percentage of the genome covered by that class of elements. **B** The percentage of eccDNA reads mapped to the specific repetitive elements. **C** and **D** The motif sequences surrounding the start and end positions of eccDNA molecules from aged and young ASCs

We further explored the genomic distribution of all identified eccDNAs by mapping to different classes of genomic elements, which included 3′ and 5′ untranslated regions (UTR), exons, introns, CpG islands, Alu elements and 2 kb regions upstream or downstream of genes. We used normalized genomic coverage to assess eccDNA distribution in these elements. The result revealed that eccDNAs in ASCs from all samples were remarkably enriched in 2 kb regions upstream and downstream of genes and were minimally enriched in CpG islands (Fig. 3A). Previous studies have demonstrated that repetitive elements are more accessible for the generation of eccDNAs. Therefore, we aligned the eccDNA reads to repetitive elements to investigate any relationship. The results showed that eccDNAs in all samples were more likely enriched in satellites and long interspersed nuclear elements (LINEs) compared with other components (Fig. 3B). No differences in genomic elements or repetitive element distributions were identified in eccDNAs from young ASC and old ASCs. The start/end site (junction) contributes to the circularization of eccDNAs. Thus, we analyzed the 10 bp sequences upstream/downstream of the junction area to investigate the potential algorithm. The results showed a pair of trinucleotide segments with 3-bp “spacers” that flanked the start and end sites of eccDNAs in ASCs from young donors (Fig. 3C). However, we did not find similar segments in ASCs from old donors (Fig. 3D).

### Differential eccDNA profiles in ASCs from young and old donors

**Fig. 4:**
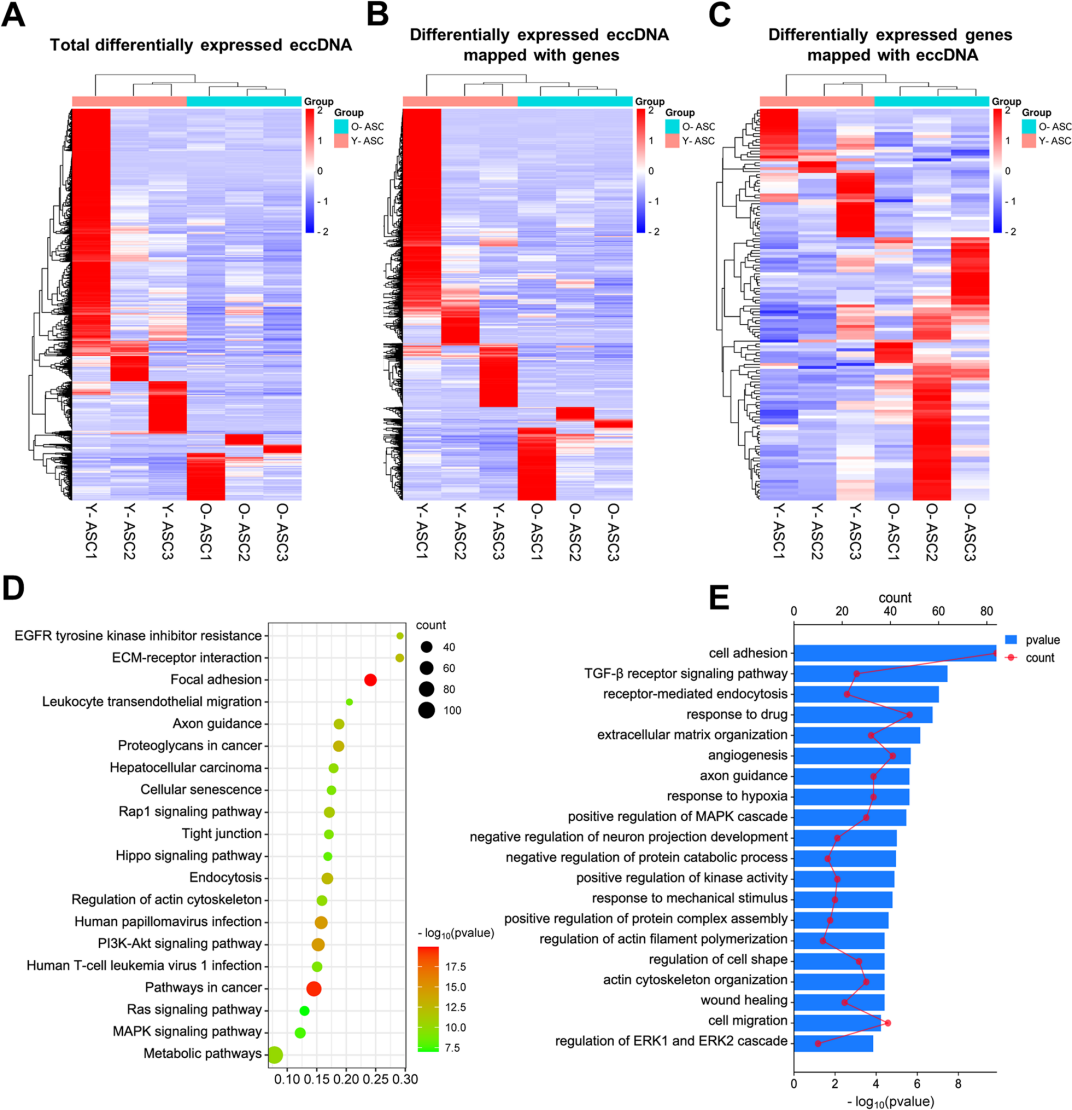
Analysis of differentially expressed eccDNAs. **A** Clustered heatmap exhibiting differentially expressed eccDNAs in aged and young ASCs. **B** Heatmap showing differentially expressed eccDNAs located within the DNA region of protein-coding genes. **C** Heatmap showing differentially expressed genes mapped to the eccDNAs. **D** KEGG analysis of the host genes associated with the differentially expressed eccDNAs. **E** GO analysis of the host genes associated with the differentially expressed eccDNAs

To explore the potential role of eccDNAs in aging of ASCs, we conducted differential expression analysis. The results showed that 3025 eccDNAs were differentially expressed, with 2514 upregulated and 511 downregulated in ASCs from young donors compared with old donors (Fig. 4A and Additional file 8: Data S7). Of them, 1932 (1472 up- and 460 downregulated) eccDNAs were annotated using GenBank (Fig. 4B and Additional file 9: Data S8), including 1488 protein-coding genes. Among these, 176 protein-coding genes were differentially expressed, including 119 upregulated and 57 downregulated genes in ASCs from young donors compared with old donors (Fig. 4C and Additional file 10: Data S9). Next, KEGG and GO analyses were performed to investigate the function of these 1488 protein-coding genes. KEGG pathway analysis revealed that 207 pathways were significantly enriched, including focal adhesion, PI3K-Akt signaling pathway, Rap1 signaling pathway, metabolic pathways, and cellular senescence (Fig. 4D). Meanwhile, GO biological process analysis showed 323 terms were statistically enriched, including cell adhesion, TGF-β receptor signaling pathway, angiogenesis, response to hypoxia, and positive regulation of the MAPK cascade (Fig. 4E).

### Conjoint analysis of differential eccDNAs and protein-coding genes in ASCs

**Fig. 5:**
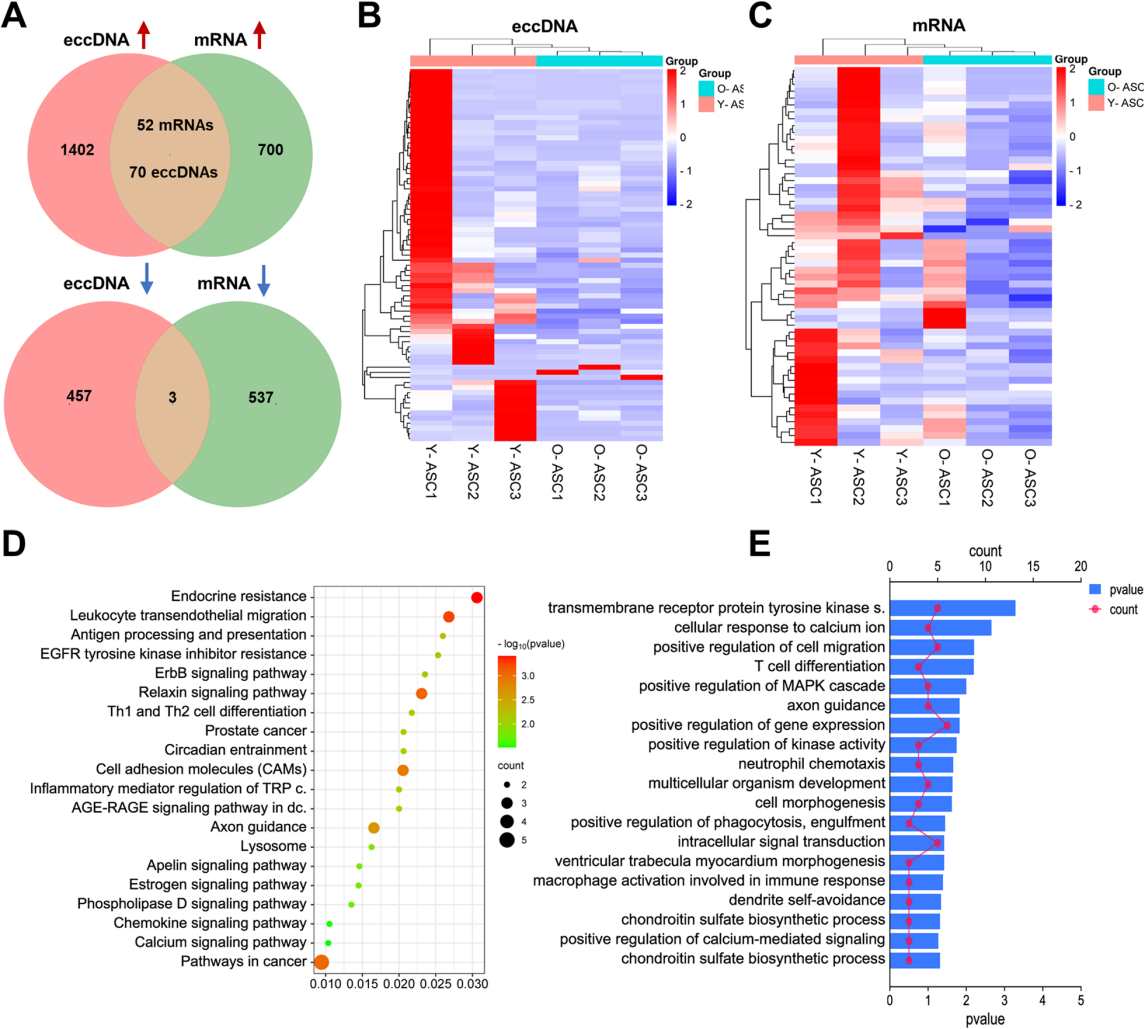
Conjoint analysis of differential eccDNAs and protein-coding genes. **A** Venn diagrams exhibiting the overlap of differentially expressed genes and eccDNAs originating from the same host genes. **B** Heatmap showing the overlapped eccDNAs. **C** Heatmap showing the overlapped genes. **D** KEGG analysis of the overlapped genes. **E** GO analysis of the overlapped genes

Our previous study identified 1292 (752 up- and 540 downregulated) differentially expressed protein-coding genes in Y-ASC compared with O-ASC by RNA-seq (Additional file 11: Data S10). We analyzed the RNA-seq results with the Circle-seq results and found that 52 mRNAs and 70 eccDNAs were overlapped and upregulated in Y-ASC compared with O-ASC, and three mRNAs and eccDNAs were overlapped and down-regulated in Y-ASC compared with O-ASC (Fig. 5A and Additional file 12: Data S11 and Additional file 13: S12). These 73 eccDNAs and 55 mRNAs were submitted for hierarchical clustering heat maps in Fig. 5B and C. We also conducted GO and KEGG analyses to investigate the biological function of these protein-coding genes. KEGG pathway analysis showed twenty-eight pathways were statistically enriched, including ErbB signaling pathway, cell adhesion molecule, estrogen signaling pathway and chemokine signaling pathway. GO biological process analysis found nineteen GO terms were significantly enriched, including transmembrane receptor protein tyrosine kinase signaling pathway, positive regulation of cell migration, positive regulation of MAPK cascade and intracellular signal transduction.

### Validations of the differentially expressed eccDNAs in ASCs

**Fig. 6:**
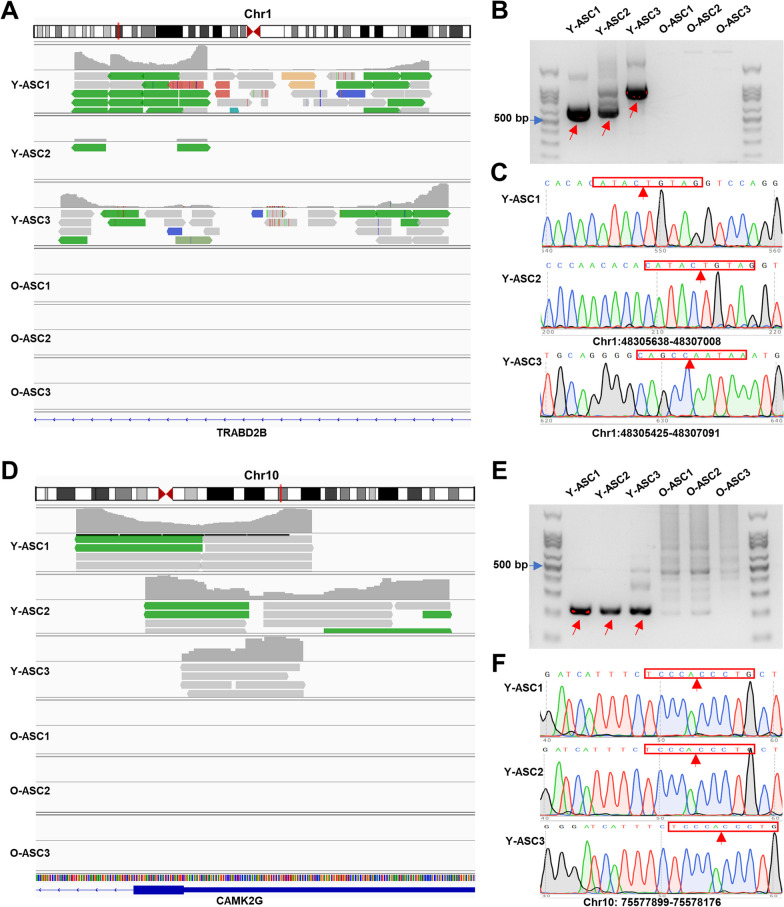
Validation of the differentially expressed eccDNAs. **A** eccDNAs derived from TRABD2B were visualized by IGV software. **B** Gel blots showing the PCR product of the identified eccDNA in each sample. **C** Sanger’s sequencing of the PCR products demonstrated the junction site of each eccDNA from TRABD2B; arrow represents the junction site. **D** eccDNAs derived from CAMK2G were visualized by IGV software. **E** Gel blots showing the PCR product of the identified eccDNA in each sample. **F** Sanger’s sequencing of the PCR products demonstrated the junction site of each eccDNA from CAMK2G; arrow represents the junction site

We next selected eccDNAs relating to TRABD2B and CAMK2G for further validation. The Circle-seq data showed that ASCs from young donors owned many eccDNA reads relating to TRABD2B, while we did not find any reads on this genome area in ASCs from old donors (Fig. 6A). Further PCR and Sanger sequencing results demonstrated that an eccDNA named TRABD2B ^(chr1: 48305638-48307008)^ existed in Y-ASC1 and Y-ASC2, another eccDNA named TRABD2B ^(chr1: 48305425-48307091)^ existed in Y-ASC3 (Fig. 6B, C). Furthermore, we also found an eccDNA named CAMK2G ^(chr10: 75577899-75578176)^ only existed in three young ASCs (Fig. 6D, E). The junction site of this eccDNA was further verified by Sanger sequencing (Fig. 6F). Importantly, we did not find any eccDNAs in ASCs from three old ASCs in these genome areas.

## Discussion

Age-related dysfunction of stem cells impairs their therapeutic capacity for organ and tissue regeneration and limits their clinical application. Novel methods are required to enrich functional stem cell subpopulations and rejuvenate aged stem cells. In this study, we broadened the current knowledge of stem cell aging at the eccDNA level and established the first eccDNA profiles of ASCs from young and old donors. Strikingly, we demonstrated three eccDNAs were depleted in old ASCs, which may be potential biomarkers for stem cell aging and promising targets for stem cell rejuvenation.

We identified hundreds of thousands of eccDNAs in ASCs, which were extensively distributed across the human genome. The number of eccDNAs was remarkably decreased in aged ASCs compared with young ASCs. This phenomenon is consistent with a previous study showing the number and heterogeneity of eccDNAs was largely diminished with age [31]. Meanwhile, another study found that eccDNA production was weakly correlated with the proliferation rate and transcriptional activity of individual tissue types [32], which is in line with our results showing that young ASCs had higher proliferative capability as demonstrated by our previous study [27]. However, the results of another study suggested that eccDNAs accumulate during aging because of increased DNA damage and decreased exclusion of nuclear eccDNAs [33–35]. Moreover, the average length of eccDNAs in young ASCs was larger than those in aged ASCs, suggesting loss of large-size eccDNAs during aging. The basic features of eccDNAs, e.g., distribution pattern and GC contents, were similar among ASC samples, which were also similar to those characterized in the plasma [36], anterior capsule [37], femoral head [38] and ovarian cancer [16]. Interestingly, unlike the typical predominant peak clusters of eccDNA size at ~ 200 bp and 350 bp [15, 28, 39], eccDNA distribution in our analysis presented only one large peak around 200 bp, reflecting a specific characteristic of eccDNAs in ASCs.

Analysis of the chromosomal distribution and genomic annotation of eccDNAs in ASCs may provide insights into the origin and generative rules of eccDNAs. In accordance with previous findings in other studies [15, 40], the majority of eccDNAs in ASCs were located in coding DNA regions and were frequently generated from chromosomes with a higher density of Alu element and protein-coding genes (e.g., chromosome 17 and chromosome 19). From the perspective of genomic elements, eccDNAs in ASCs were remarkably enriched in 2 kb regions upstream/downstream of genes and minimally enriched in CpG islands, which was similar to those previously identified in plasma and breast cancer [36, 41]. These results suggest that the formation of eccDNAs is associated with chromatin accessibility, which also aligns with previous findings that showed eccDNA formation was correlated with transcriptional activity [32]. Furthermore, previous studies revealed repetitive regions are more likely to form eccDNAs [42]. Similar results have been found in our study; eccDNAs in ASCs were frequently distributed in satellites and LINEs, which is similar to the eccDNAs in ovarian cancer cells [16]. The sequences around the junction sites contribute to the circulation of eccDNAs. Previous studies focused on these sequences and found a pair of trinucleotide segments with 4-bp “spacers” flanking the junction sites [15, 28]. In this study, similar trinucleotide motifs were identified in eccDNAs from young donors, suggesting their widespread existence in eccDNAs from different cells and vital roles in eccDNA generation. However, similar trinucleotide motifs were not identified in eccDNAs from old donors, suggesting aging diminished lots of eccDNAs generated according to this rhythm.

eccDNAs have been particularly well studied in cancers. Little is known about the role of eccDNAs in stem cell maintenance, differentiation or aging. In our study, we found that thousands of eccDNAs were differentially expressed in ASCs from young donors compared with old donors, and the host genes of some eccDNAs exhibited the same differential expression between the two groups. Further, GO and KEGG analyses of differentially expressed eccDNAs indicated that cellular senescence, cell adhesion and TGF-β receptor signaling pathways were statistically enriched, which have been previously shown to be been associated with stem cell aging in other RNA-seq studies [27, 43, 44]. The function of eccDNAs remains largely unknown. Previous studies demonstrated that eccDNAs could encode oncogenes and miRNAs [40, 45, 46], promote accessible chromatin [47], and function as enhancers for tumor progression [48]. To further understand the roles of eccDNAs in ASCs, differentially expressed eccDNAs were selected for further validation. We found three eccDNAs could only be detected in young ASCs. CAMK2G ^(chr10: 75577899-75578176)^ is generated from CAMK2G, which is one of the four subunits of serine/threonine kinases that mediate the second messenger effects of Ca^2+^. TRABD2B ^(chr1: 48305638-48307008)^ and TRABD2B ^(chr1: 48305425-48307091)^ are generated from TRABD2B, which functions as a metalloprotease regulating the Wnt signaling pathway. These eccDNAs are lost during adipose stem cell aging, which is in line with a previous study on loss of eccDNA diversity in aged yeast cells [31]. In contrast to long-length eccDNAs carrying whole genes or their multiple copies in cancer cells, these three eccDNAs are small in size and lack obvious transcriptional potential. Further studies are required to investigate the function of these small eccDNAs, especially in stem cells during aging.

## Conclusion

In the present study, we identified and characterized eccDNAs in ASCs from young and old donors by Circle-seq. We identified thousands of eccDNAs and found that basic characteristics such as high GC contents, size distribution, genomic annotation and junction motif were similar with previously reported eccDNAs. Moreover, we identified thousands of differentially expressed eccDNAs, most of which were generated from genome areas containing protein-coding genes, which were further analyzed by GO and KEGG databases. Importantly, we verified three eccDNAs including CAMK2G ^(chr10: 75577899-75578176)^, TRABD2B ^(chr1: 48305638-48307008)^ and TRABD2B ^(chr1: 48305425-48307091)^ were only detected in young ASCs. These eccDNAs were depleted in old ASCs, suggesting their potential roles in stem cell aging and as novel targets for stem cell rejuvenation.

### Supplementary Information

**Additional file 1: Table S1.** Basic characteristics of different groups. **Table S2.** Primers used for polymerase chain reaction. **Fig. S1.** Original blots.

**Additional file 2: Data S1.** eccDNAs detected in Y-ASC1.

**Additional file 3: Data S2.** eccDNAs detected in Y-ASC2.

**Additional file 4: Data S3.** eccDNAs detected in Y-ASC3.

**Additional file 5: Data S4.** eccDNAs detected in O-ASC1.

**Additional file 6: Data S5.** eccDNAs detected in O-ASC2.

**Additional file 7: Data S6.** eccDNAs detected in O-ASC3.

**Additional file 8: Data S7.** Differentially expressed eccDNAs.

**Additional file 9: Data S8.** Differentially expressed eccDNAs annotated using GenBank.

**Additional file 10: Data S9.** Differentially expressed genes mapped with differentially expressed eccDNAs.

**Additional file 11: Data S10**. Differentially expressed genes.

**Additional file 12: Data S11.** Overlapped eccDNAs.

**Additional file 13: Data S12.** Overlapped genes.

## Data Availability

All data generated and/or analyzed during this study are available from the corresponding author upon reasonable request. The RNA expression profiles were retrieved from the Gene Expression Omnibus (GEO) database (https://www.ncbi.nlm.nih.gov/geo/). The GEO series accession number is GSE174502. The raw sequence data of eccDNAs reported in this paper have been deposited in the Genome Sequence Archive in National Genomics Data Center, China National Center for Bioinformation/Beijing Institute of Genomics, Chinese Academy of Sciences (GSA-Human: HRA005358) that are publicly accessible at https://ngdc.cncb.ac.cn/gsa-human.

## Abbreviations

ASC: Adipose stem cell
eccDNA: Extrachromosomal circular DNA
PCR: Polymerase chain reaction
CAM2KG: Calcium/calmodulin-dependent protein kinase II gamma
TRABD2B: TraB domain containing 2B
Gene2kbD: 2 Kb downstream of genes
Gene2kbU: 2 Kb upstream of genes
tDNA: Transport DNA repeats
srpDNA: Signal recognition particle DNA repeats
snDNA: Small nuclear DNA repeats
SINE: Short interspersed nuclear element
scDNA: Small conditional DNA repeats
rDNA: Ribosomal DNA repeats
RC: Rolling circle repeats
LTR: Long terminal repeat
LINE: Long interspersed nuclear element
