# Supplementary material for: Deciphering the role of extrachromosomal circular DNA in adipose stem cells from old and young donors
Source: Stem Cell Res Ther. 2023 Nov 28;14:341. doi: 10.1186/s13287-023-03575-2 (PMC10683086; doi:10.1186/s13287-023-03575-2)
Supplement: Supplementary file 1 — Additional file 1: Table S1. Basic characteristics of different groups. Table S2. Primers used for polymerase chain reaction. Fig. S1. Original blots. [file 13287_2023_3575_MOESM1_ESM.docx]

| Variables | Young donors | Old donors | P value (Y/O) |
| --- | --- | --- | --- |
| Number | 3 | 3 | NA |
| Age  Range | 10.33 ± 0.47  [10, 11] | 57.33 ± 2.62  [55, 61] | <0.001***  NA |
| BMI | 21.05 ± 2.45 | 21.77 ± 1.94 | 0.76 |
| SBP  DBP | 114.67 ± 5.25  76.00 ± 5.89 | 133.67 ± 13.42  84.00 ± 8.64 | 0.14  0.34 |
| FBG  Cr  BUN | 5.24 ± 0.08  37.87 ± 4.42  3.96 ± 0.20 | 5.13 ± 0.56  61.13 ± 1.11  4.26 ± 0.53 | 0.78  0.002**  0.50 |

Table S1: Basic characteristics of different groups included in the study.

Range: minimum age, maximum age; BMI (kg/m^2^): body mass index; SBP (mm Hg): systolic blood pressure; DBP (mm Hg): diastolic blood pressure; FBG (mmol/L): fasting blood glucose; Cr (μmol/L): creatinine; BUN (mmol/L): blood urea nitrogen; NA: not available. Data are expressed as Mean ± SD and were statistically analyzed by the un-paired Student’s t test. *p< 0.05, **p< 0.01, ***p< 0.001.

| eccDNA | Primer sequences |
| --- | --- |
| eccDNA-CAMK2G  eccDNA-TRABD2B | Forward 5’-ACCAGACAGGGCTCAGACT-3’’  Forward 5’-GAGAGGTAAATGGAGCAGC-3’’  Forward 5’-AGGAATCAACTTGGGGCG-3’’  Forward 5’-GTAAACACTCTCCAGGAACTCAC-3’’ |

Table S2: Primers used for polymerase chain reaction.

Figure S1: Original blots

**O-ASC3**

**O-ASC1**

**O-ASC2**

**Y-ASC3**

**Y-ASC2**

**Y-ASC1**


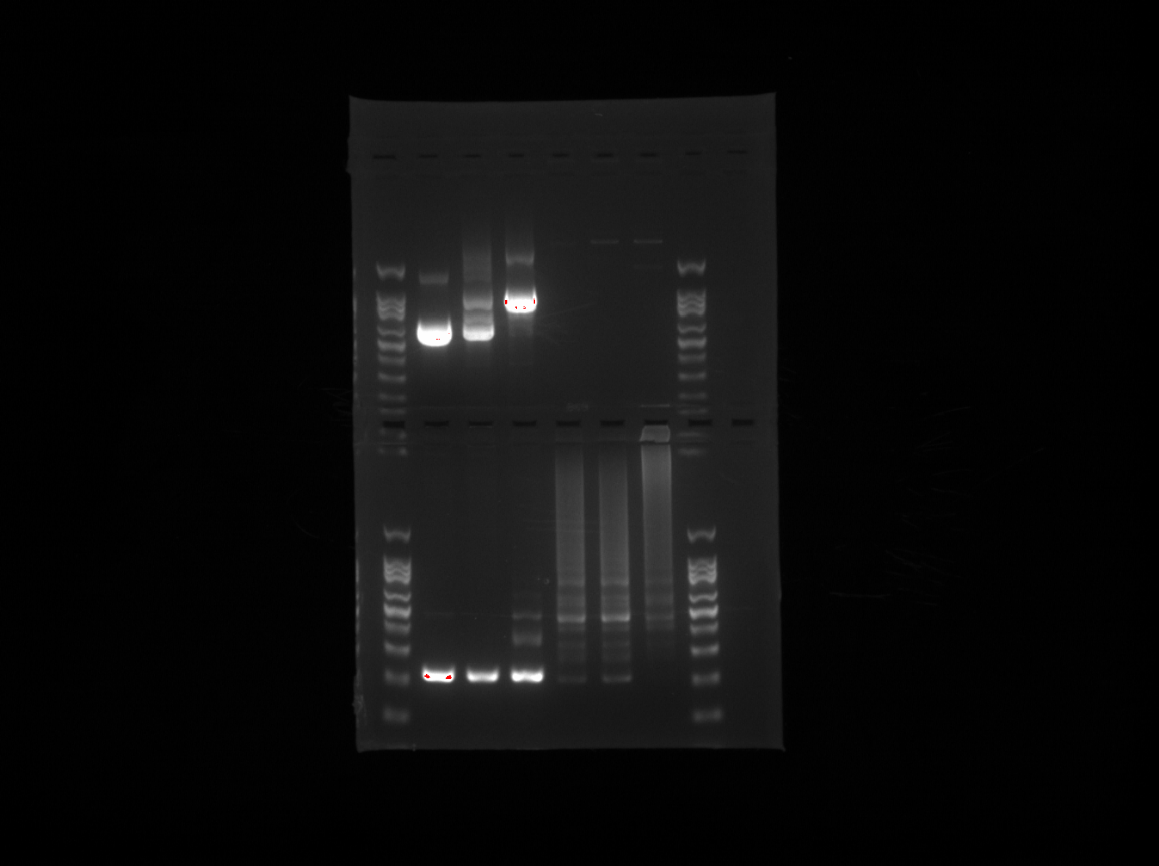


Figure 6E

Figure 6B
